# Role of Intracellular Ca^**2+**^ and Na^**+**^/Ca^**2+**^ Exchanger in the Pathogenesis of Contrast-Induced Acute Kidney Injury

**DOI:** 10.1155/2013/678456

**Published:** 2013-11-18

**Authors:** Dingping Yang, Dingwei Yang

**Affiliations:** ^1^Division of Nephrology, Department of Internal Medicine, Renmin Hospital of Wuhan University, Wuhan 430060, China; ^2^Division of Nephrology, Department of Internal Medicine, General Hospital of Tianjin Medical University, Tianjin 300052, China

## Abstract

The precise mechanisms underlying contrast-induced acute kidney injury (CI-AKI) are not well understood. Intracellular Ca^2+^ overload is considered to be a key factor in CI-AKI. Voltage-dependent Ca^2+^ channel (VDC) and Na^+^/Ca^2+^ exchanger (NCX) system are the main pathways of intracellular Ca^2+^ overload in pathological conditions. Here, we review the potential underlying mechanisms involved in CI-AKI and discuss the role of NCX-mediated intracellular Ca^2+^ overload in the contrast media-induced renal tubular cell injury and renal hemodynamic disorder.

## 1. Pathogenesis of CI-AKI

Contrast-induced acute kidney injury (CI-AKI) is the third leading cause of hospital-acquired acute renal failure accounting for 10–12% of all causes of hospital-acquired renal failure [1]. In general population, the incidence is 1–6%. In some special populations, such as patients with underlying hypertension, cardiovascular diseases, diabetes mellitus, or preexisting renal insufficiency, the incidence is higher and may be as high as 20–50% [2–4]. In patients undergoing coronary angiography in China, the incidence of CI-AKI is 8.7%–23.5% [5, 6]. The precise mechanisms underlying CI-AKI are not fully understood, especially its cellular and molecular mechanism. But, it is clear that disturbance of renal hemodynamics and direct toxic action on renal tubular cells are main factors responsible for CI-AKI. Previous investigations [7, 8] have shown that contrast media administration can result in initial renal vasodilatation (about 20 minutes), followed by prolonged vasoconstriction (about 20 minutes to several hours). Subsequent studies [9, 10] demonstrated that there were regional differences in the vascular response to contrast media, with a greater reduction in flow to the outer medulla. And now, it has been verified that contrast-induced selective reduction in renal medullary blood flow and the secondary hypoxia in this region is a major underlying cause of CI-AKI [10]. It has been reported that calcium channel blockers (CCB) can reverse the acute hemodynamic alterations induced by contrast administration and alleviated CI-AKI [11–13]. Furthermore, our experimental animal investigation [14] also verified that tail vein injection of an inhibitor of reverse mode of Na^+^/Ca^2+^ exchanger (NCX) can suppress the contrast-induced ET-1 overproduction and renal vasoconstriction. These findings suggested that intracellular Ca^2+^ overload plays an important role in contrast-induced renal hemodynamic disorder. Besides changes in calcium physiology, contrast-induced vasoconstriction might also be a result of a direct effect on vascular smooth muscle [15] or from a local increase in adenosine [16] and endothelin [17] production.

It must be pointed out that, under normal circumstances, the contrast-induced renal hemodynamic disorder was not enough to induce CI-AKI based on the facts that humans as well as experimental animals without risk factors do not usually exhibit CI-AKI following contrast media injection. This is because, under physiological state, the renal circulation is subjected to autoregulation which is associated with neural, hormonal, paracrine, and autocrine influences. Injured autoregulation of microcirculation might be the cause that all kinds of risk factors such as preexisting renal impairment, diabetes mellitus, and hypercholesterolemia,   make the kidney vulnerable to iodinated contrast media.

Renal tubular cells apoptosis is a key mechanism of CI-AKI. Studies have shown that contrast media can induce renal tubular epithelial cell apoptosis via ROS (reactive oxygen species) pathway, JNK/p38 stress kinase pathway, and intrinsic apoptotic pathways [18–20] and can also result in renal tubular epithelial cell injury by dephosphorylation (inactivation) of the kinase Akt [21]. But it is still unclear why contrast media can cause ROS overproduction and why contrast media can activate p38 Mitogen-Activated Protein Kinases (MAPK). Our recent studies showed that contrast-induced ROS overproduction, p38 activation, and tubular cell apoptosis might be associated with intracellular calcium overload [19, 22, 23].

## 2. The Role of Intracellular Ca^**2+**^ in the Pathogenesis of Contrast-Induced Acute Kidney Injury

Intracellular calcium overload is considered to be a key factor in ischemic cell injury and CI-AKI [12]. Studies have shown that both renal vasoconstriction and renal tubular apoptosis induced by contrast media are associated with changes in calcium physiology [11, 13, 22, 23]. Although physiological and pathophysiological mechanisms of Ca^2+^ overload in ischemic kidney and CI-AKI have not been fully elucidated, there is evidence indicating that increased cytosolic Ca^2+^ may be an important mediator of epithelial cell apoptosis and necrosis [24]. So, theoretically, CCB would have protective effects on CI-AKI. In clinical practice, CCB can reverse the acute hemodynamic alterations induced by radiocontrast administration and alleviated CI-AKI [11–13]. However, acute administration of CCB before contrast media administration is not enough to prevent CI-AKI [25]. Only one small trial demonstrated any value with CCB [13] whereas other studies showed no beneficial effects [26, 27]. The fact that acute administration of CCB before contrast media administration was not enough to prevent CI-AKI suggested that the intracellular Ca^2+^ overload induced by contrast media might not be completely suppressed by CCB. So we cannot conclude based on these clinical data that intracellular calcium overload was not associated with CI-AKI because VDC is not the only pathway that induces Ca^2+^ influx. There is the possibility that other channels besides VDC may also be involved in the contrast-induced intracellular calcium overload. Our recent study [22] has shown that contrast media resulted in NRK-52E cell apoptosis via the induction of an increase in intracellular Ca^2+^ and reactive oxygen species and KB-R7943, inhibitor of the reverse mode of NCX, attenuated the contrast media-induced renal tubular epithelial cell apoptosis by suppressing the intracellular Ca^2+^ overload and reducing oxidative stress, which suggested that intracellular Ca^2+^ overload via the NCX system is also involved in contrast-induced renal tubular apoptosis.

## 3. The Role of Na^**+**^/Ca^**2+**^ Exchanger System in the Pathogenesis of CI-AKI

NCX is a bidirectional plasma membrane transporter that catalyzes the exchange of 3 or 4 Na^+^ for 1 Ca^2+^, depending on the electrochemical gradients of the substrate ions [28, 29] and is encoded by a multigene family comprising 3 NCX isoforms: NCX1, which is expressed in various organs including the kidney [30]; and NCX2 and NCX3, which are expressed mainly in the brain and skeletal muscle [31, 32]. Under physiological conditions, NCX can pump the Ca^2+^ outside the cell using the Na^+^ concentration gradient across the cell membrane to keep a low intracellular Ca^2+^ level, which is referred to as the forward-mode operation of the exchanger. In pathological conditions, NCX can reversely extrude Na^+^ for Ca^2+^ influx and result in intracellular Ca^2+^ overload, which is referred to as the reverse mode or calcium influx mode of NCX. In the normal kidney, NCX plays an important role in the active calcium transport in distal convoluted tubules [33]. In the ischemia-reperfusion kidney and in the hypoxia-reoxygenation renal tubular epithelial cells, NCX reversely extrudes Na^+^ for Ca^2+^ influx and results in intracellular Ca^2+^ overload and tubular epithelial cell injury [34, 35].

It has been verified that contrast media can induce renal tubular epithelial cell apoptosis via ROS pathway, JNK/p38 pathway, and intrinsic apoptosis pathway [18, 20]. Our recent in vitro studies [19, 36] demonstrated that contrast-induced ROS overproduction, p38 activation, and apoptosis in renal tubular cell were associated with the increase of intracellular Ca^2+^. The inhibitor of reverse mode of NCX, KB-R7943, can alleviate contrast-induced renal tubular apoptosis through suppressing the increase of intracellular Ca^2+^ and subsequent ROS overproduction and p38 activation. These data demonstrate that intracellular Ca^2+^ overload via the reverse mode of NCX system is involved in contrast-induced renal tubular epithelial cell apoptosis.

Recent animal model experiments [14, 37] also showed that pretreatment with tail vein injection of KB-R7943 markedly and dose-dependently suppressed the increase in renal ET-1 production and the reduction in renal blood flow induced by contrast medium administration and prevented contrast-induced acute renal failure, which suggested that Ca^2+^ overload via the reverse mode of NCX, followed by renal ET-1 overproduction and renal vasoconstriction, plays an important role in the pathogenesis of CI-AKI.

## 4. Hypothesis about the Molecular Mechanism of CI-AKI

**Figure 1:**
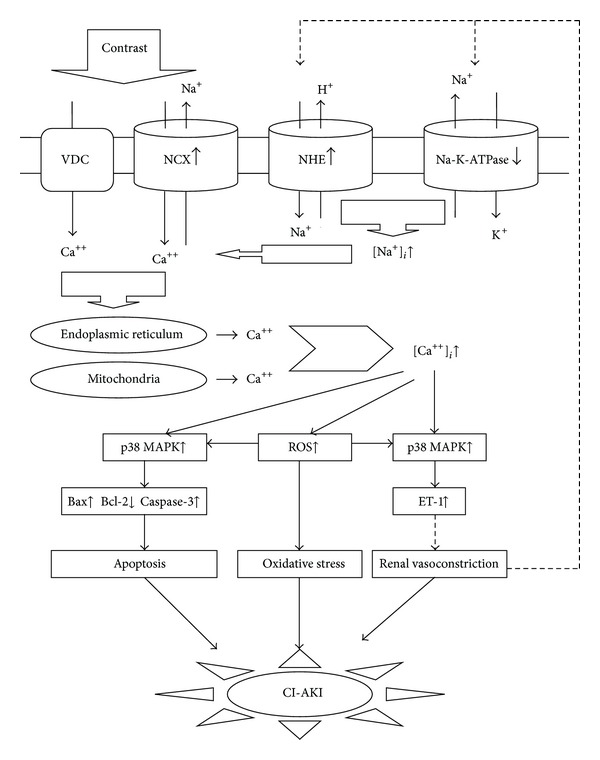
The diagram shows the proposed molecular mechanism of CI-AKI. CI-AKI, contrast-induced acute renal injury; NCX, Na^+^/Ca^2+^ exchanger; VDC, the voltage-dependent Ca^2+^ channel; NHE, Na^+^/H^+^ exchange; [Ca^++^]_*i*_, intracellular Ca^2+^ concentration; [Na^+^]_*i*_, intracellular Na^+^ concentration; p38 MAPK (p38 Mitogen-Activated Protein Kinases); ROS, reactive oxygen species. ET-1, endothelin-1; ATP, adenosine triphosphate.

Based on the findings [19, 22, 36] that inhibition of the reverse mode of NCX alleviated contrast-induced renal tubular cell apoptosis through suppressing the increase of intracellular Ca^2+^, ROS overproduction, p38 MAPK activation, and Caspase-3 overexpression and the findings [14, 37] that tail vein injection of inhibitor of reverse mode of NCX can exert protective effects on CI-AKI in rats through suppressing contrast-induced renal ET-1 overproduction and renal vasoconstriction, we propose the following hypothesis regarding the molecular mechanism of CI-AKI. Contrast medium exposure activates the reverse mode of NCX1 expressed in renal tubular epithelial cells; NCX reversely extrudes Na^+^ for Ca^2+^ influx and results in increased intracellular Ca^2+^. The increased intracellular Ca^2+^ can stimulate Ca^2+^ release from the mitochondrial and endoplasmic reticulum and result in intracellular Ca^2+^ overload [38]. The intracellular Ca^2+^ overload via the reverse mode of NCX and VDC induced by contrast media in the renal tubular epithelial cell can result in ROS overproduction and oxidative stress. Increased ROS and intracellular Ca^2+^ can induce upregulation of p38 and p-p38 MAPK expression [36] and subsequently activate intrinsic apoptotic pathways such as bcl-2, bax, and caspase-3 and result in renal tubular epithelial cell apoptosis, which is the underlying cause of contrast-induced direct renal tubular toxicity. p38 MAPK activation via the reverse mode of NCX and VDC could also result in renal ET-1 overproduction, followed by renal vasoconstriction and renal ischemia, which is one of the underlying causes of contrast-induced renal hemodynamic abnormalities. ET-1 overproduction and renal ischemia can cause depletion of adenosine triphosphate (ATP) and development of intracellular acidosis. The accumulation of intracellular Na^+^, which is caused by inhibition of Na^+^/K^+^-ATPase activity because of decreased ATP production [39] and activation of the Na^+^/H^+^ exchange because of intracellular acidosis [40], can also activate the reversion of the mode of NCX and subsequently cause calcium overload and ET-1 overproduction, forming a vicious cycle. The diagram of the hypothesis about the molecular mechanism of CI-AKI is seen in Figure 1. Contrast media exposure activates VDC and the reverse mode of NCX expressed in the renal tubular epithelial cell and induces Ca^2+^ influx. The increased intracellular Ca^2+^ stimulates Ca^2+^ release from the mitochondrial and endoplasmic reticulum and results in intracellular Ca^2+^ overload, which induced ROS overproduction and oxidative stress. Increased ROS and intracellular Ca^2+^ activate p38 MAPK. On one hand, p38 MAPK activates intrinsic apoptotic pathways such as bcl-2, bax, and caspase-3 and induces renal tubular epithelial cell apoptosis, which is the underlying cause of contrast-induced direct renal tubular toxicity. On the other hand, activated p38 MAPK also results in renal ET-1 overproduction, followed by renal vasoconstriction and renal ischemia, which is one of the underlying causes of contrast-induced renal hemodynamic abnormalities. ET-1 overproduction and renal ischemia can cause depletion of ATP and development of intracellular acidosis, which can result in accumulation of intracellular Na^+^ and further activate the reversion of the mode of NCX and subsequently cause Ca^2+^ influx and ET-1 overproduction, forming a vicious cycle.

## 5. Conclusion

In summary, Ca^2+^ overload via the reverse mode of NCX1 and VDC, followed by ROS overproduction, p38 MAPK activation, and ET-1 overproduction, plays an important role in the contrast-induced renal hemodynamic disorder and renal tubular epithelial cell apoptosis, which suggests that, in clinical practice, CCB should be recommended to patients with hypertension who are undergoing radiographic examination or therapy requiring contrast media and that selective inhibitors of NCX1 may be beneficial in the prevention and treatment of CI-AKI in humans.
